# National Association of Dental Examiners

**Published:** 1894-11

**Authors:** 


ARTICLE II.

### NATIONAL ASSOCIATION OF DENTAL EXAMINERS.

The thirteenth annual meeting of the National Association of Dental Examiners was held at Old Point Comfort, Va., beginning Tuesday, August 7, 1894; Dr. J. Searle Hurlbut, presiding. In the absence of the Secretary, Dr. C. A. Meeker was appointed Secretary *pro tem.*

The following State boards were represented: Alabama, T. P. Whitby; Delaware, D. M. Hitch, C. R. Jefferis; District of Columbia, H. B. Noble, Wm. Donnally; Georgia, B. H. Catching; Illinois, L. L. Davis; Iowa, J. T. Abbott; Kansas, L. C. Wasson; Kentucky, C. G. Edwards; Massachusetts, J. Searle Hurlbut; New Jersey, F. C. Barlow, Chas. A. Meeker; North Carolina, V. E. Turner; Pennsylvrnia, W. E. Magill, Louis Jack, L. Ashley Faught; Tennessee, J. Y. Crawford; Virginia, J. Hall Moore, E. P. Beadles.

The Secretary read a communication from the board of examiners of the State of Minnesota, insisting that their resignation, tendered in 1892, should be accepted. On motion of Dr. C. G. Edwards, the resignation was accepted.

An application for membership from the board of examiners of the State of Oregon was received and the Secretary instructed to answer the same.

On motion of Dr. F. C. Barlow, the Secretary was instructed to have the constitution, by-laws and standing resolutions to date printed in pamphlet form and a copy sent to all members of the association.

On motion of Dr. Louis Jack, the secretary was instructed to have the proceedings of the association at its meetings of 1893-4 printed in one pamphlet.

The Secretary read a memorial from the examining boards of New York, New Jersey, Delaware, Rhode Island, Connecticut, Pennsylvania and District of Columbia, regarding the necessity of uniformity in standards of examinations in different States, which, with the following resolution on the same subject, laid over last year, was referred to a committee consisting of Drs. Faught, Crawford and Catching:

"*Resolved*, That it is the sense of the National Association of Dental examiners, that when a member of the dental profession presents a certificate of registration from a State board of dental examiners, duly created by law, that the same should entitle the holder of such certificate to registration without an additional examination in any State of the Union having a law to regulate the practice of dentistry. *Provided*, such certificate was obtained on examination."

The committee subsequently reported, recommending the adoption of the memorial as expressing the sense of the association.

The report was adopted, a verbal amendment to the memorial offered by Dr. Donnally being accepted, making it read as follows:

"*To the National Association of Dental Examiners*:

"GENTLEMEN,—Delegates from the examining boards of New York, New Jersey, Delaware, Rhode Island, District of Columbia, Pennsylvania and Connecticut, in conference assembled, respectfully offer for your consideration the opinion that ours is an advancing profession, our watchword is progress, and we ask that your influence shall be actively directed to secure thorough preliminary examinations in every dental college in every State.

"We think the true interests of our profession demand such uniformity in standards of examinations as can be obtained by State boards, always striving after higher and better attainments, and with a view to ultimately bring about a safe, judicious and fair reception of certificates from State boards of other states.

"It is our opinion that it would be most desirable that a law whose provisions should be uniform, yet whose phraseology might be different, so as to suit differences of environment in the various States, should be enacted in all the States. It is not practicable to have any such law enacted in all the States at once; but if such a law were enacted in half a dozen leading States, other States would gradually fall into line and adopt similar ones.

"We are in favor of bringing responsibility for defective education as directly as possible upon the offending school, with a view to making every diploma real and reliable evidence as regards the holder's ability and proficiency. Therefore we will use our influence as practitioners, as well as members of examining boards, to obtain the selection, by competent authority, in each State, of an independent body, disconnected from the institutions which educate, with sole authority to grant licenses and admit to practice.

"G. CARLETON BROWN, *Secretary*."

The Committee on Colleges reported that there are now twenty-eight recognized and fifteen unrecognized colleges; that there has been no addition to the accepted schools.

The chairman, Dr. Jack, read the following report of the numbers of matriculates and graduates at the different schools:

| Recognized Schools—1894. | Freshmen. | Juniors. | Seniors. | Graduates. | Post Graduates. |
|---|---|---|---|---|---|
| 1. Baltimore College of Dental Surgery, Md | 47 | 45 | 40 | 37 | ... |
| 2. Boston Dental College, Mass | 55 | 55 | 35 | 33 | ... |
| 3. Chicago College of Dental Surgery, Ill | 127 | 116 | 69 | 58 | 28 |
| 4. College of Dentistry, Department of Medicine, University of Minnesota | 23 | 12 | 6 | 6 | .... |
| 5. Dental Department, Columbian University, D. C. | 12 | 19 | 8 | 8 | 1 |
| 6. Dental Department National University, D. C | 6 | 9 | 8 | 8 | .... |
| 7. Northwestern University Dental School, Ill | 33 | 36 | 25 | 24 | 6 |
| 8. Dental Department Southern Medical College. Ga.* | | ... | ... | ... | ... |
| 9. Dental Department University of Tennessee | 16 | 12 | 12 | 10 | ... |
| 10. Dental Department Harvard University, Mass | 31 | 14 | 18 | 14 | ... |
| 11. Indiana Dental College Ind | 29 | 32 | 23 | 23 | ... |
| 12. Kansas City Dental College, Mo. | 54 | 36 | 18 | 16 | ... |
| 13. Louisville College of Dentistry, Ky. | 54 | 27 | 10 | 9 | ... |
| 14. Missouri Dental College | 41 | 31 | 21 | 21 | ... |
| 15. New York College of Dentistry | 111 | 92 | 81 | 62 | 1 |
| 16. Northwestern College of Dental Surgery, Ill | 32 | 36 | 25 | 24 | ... |
| 17. Ohio College of Dental Surgery | 57 | 57 | 34 | 34 | ... |
| 18. Pennsylvania College of Dental Surgery | 100 | 80 | 63 | 62 | ... |
| 19. Philadelphia Dental College | 93 | 107 | 78 | 77 | ... |
| 20. School of Dentistry of Meharry Medical Department of Central Tennessee College | 4 | 3 | 3 | 3 | ... |
| 21. Dental Department University of California | 68 | 45 | 18 | 18 | ... |
| 22. Dental Department University of Iowa | 70 | 39 | 32 | 31 | 1 |
| 23. University of Maryland, Dental Department | 54 | 44 | 36 | 34 | 2 |
| 24. University of Michigan, Dental Department | 67 | 52 | 66 | 64 | 1 |
| 25. University of Pennsylvania, Dental Department | 81 | 72 | 65 | 64 | 3 |
| 26. Vanderbilt University, Tenn | 55 | 44 | 25 | 25 | ... |
| 27. Western Dental College, Mo | 42 | 36 | 27 | 26 | ... |
| 28. American College of Dental Surgery, Ill | 128 | 71 | 51 | 44 | 77 |

*No report.

| UNRECOGNIZED SCHOOLS. | FRESHMEN. | JUNIORS. | SENIORS. | GRADUATES. | POST GRADUATES. |
|---|---|---|---|---|---|
| University of Denver, Dental Department | 9 | 4 | 3 | 3 | ... |
| University of Buffalo, Dental Department | 38 | 32 | 6 | 6 | ... |
| Cincinnati College of Dental Surgery. | 5 | 3 | 5 | 5 | ... |
| Atlanta Dental College | 61 | 31 | 26 | 26 | 8 |
| Howard University, Dental Department | 6 | 6 | 5 | ... | |
| Dental Department Homœopathic Hospital College | 12 | 7 | 4 | 4 | ... |
| Western Reserve University, Dental Department | 9 | 15 | 4 | 4 | ... |
| Cincinnati College of Medicine and Surgery, Dental Department.* | ... | ... | ... | ... | ... |
| Detroit College of Medicine, Department of Dentistry | 16 | 21 | 1 | 1 | ... |
| University College of Medicine, Department of Dentistry, Richmond, Va.* | ... | ... | ... | ... | ... |
| The New York Dental School.* | ... | ... | .. | ... | ... |
| Birmingham Dental College, Alabama. | 17 | 7 | 3 | 3 | ... |
| Marion-Sims College of Medicine, Dental Department, St. Louis (first session, Sept. 11. 1894.* | ... | ... | ... | ... | ... |
| Dental Department, Tennessee Medical College.* | ... | ... | ... | ... | ... |
| United States Dental College, Chicago* | ... | ... | ... | ... | ... |

*No Report.

The committee recommended the adoption and publication by the board of the following resolution:

"*Resolved*, That it is the sense of this board that the Kansas City College of Dental Surgery is not only not worthy of recognition, but that we have conclusive evidence that it is a fraudulent concern. And further, we would warn the public to be on their guard, because of the similarity of title with a reputable school of the same city."

The resolution was adopted.

On motion of Dr. Meeker, the following was adopted:

"*Resolved*, That any State board not represented by delegates at three successive meetings of this body should forfeit its membership."

On motion of Dr. Louis Jack, the following was adopted.

"*Resolved*, That this body require the board of examiners in each State to become informed of the character of any school which may have been organized within its jurisdiction, and to have especial care in its scrutiny of any college which may have applied for admission to the National Association of Dental Faculties."

The following were elected officers for the ensuing year:

L. Ashley Faught, President; J. T. Abbott, Vice-President; Chas. A. Meeker, Secretary and Treasurer.

After which the association adjourned to meet at the time and place appointed for the next annual meeting of the American Dental Association, this body to convene at 10 a. m. on the day preceding the date set for the meeting of the American Dental Association.

